# EBV‐LMP1 induces APOBEC3s and mitochondrial DNA hypermutation in nasopharyngeal cancer

**DOI:** 10.1002/cam4.3357

**Published:** 2020-08-20

**Authors:** Kousho Wakae, Satoru Kondo, Hai Thanh Pham, Naohiro Wakisaka, Lusheng Que, Yingfang Li, Xin Zheng, Kento Fukano, Kouichi Kitamura, Koichi Watashi, Hideki Aizaki, Takayoshi Ueno, Makiko Moriyama‐Kita, Kazuya Ishikawa, Yosuke Nakanishi, Kazuhira Endo, Masamichi Muramatsu, Tomokazu Yoshizaki

**Affiliations:** ^1^ Department of Molecular Genetics Graduate School of Medical Science Kanazawa University Kanazawa Japan; ^2^ Department of Virology II National Institute of Infectious Diseases Tokyo Japan; ^3^ Division of Otorhinolaryngology and Head and Neck Surgery Kanazawa University Kanazawa Japan; ^4^ Department of Virology II National Institute of Infectious Diseases Musashi‐Murayama Tokyo Japan

**Keywords:** APOBEC, EBV‐LMP1, mitochondrial DNA, nasopharyngeal cancer

## Abstract

An Epstein‐Barr virus (EBV)—encoded latent membrane protein 1 (LMP1) is a principal oncogene that plays a pivotal role in EBV‐associated malignant tumors including nasopharyngeal cancer (NPC). Recent genomic landscape studies revealed that NPC also contained many genomic mutations, suggesting the role of LMP1 as a driver gene for the induction of these genomic mutations. Nonetheless, its exact mechanism has not been investigated. In this study, we report that LMP1 alters the expression profile of APOBEC3s(A3s), host deaminases that introduce consecutive C‐to‐U mutations (hypermutation). In vitro, LMP1 induces APOBEC3B (A3B) and 3F(A3F), in a nasopharyngeal cell line, AdAH. Overexpression of LMP1, A3B, or A3F induces mtDNA hypermutation, which is also detectable from NPC specimens. Expression of LMP1 and A3B in NPC was correlated with neck metastasis. These results provide evidence as to which LMP1 induces A3s and mtDNA hypermutation, and how LMP1 facilitates metastasis is also discussed.

## Graphical abstract

EBV LMP1 induces host cytidine deaminases, APOBECs, in nasopharyngeal cells. TES2 domain is important for LMP1‐induced LMP1 hypermutates host mitochondrial genome.

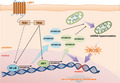

## 1 INTRODUCTION

Epstein‐Barr virus (EBV) is a cause for many types of cancers including lymphoma and nasopharyngeal cancers (NPC).^1^ It infects the tonsillar B cells and is transmitted to nasopharyngeal epithelium. In these EBV‐associated tumors, infection is latent, and only a part of the whole EBV genome is expressed, including six EBV nuclear antigens (EBNA1, −2, −3A, −3B, −3C, and ‐LP), three latent membrane proteins (LMP1, −2A, ‐and −2B), and two small RNAs (EBER1 and −2).

Among the latent viral genes described above, LMP1 immortalizes B lymphocytes and rodent epithelial cells, and is recognized as a principal oncogene that plays a pivotal role in EBV‐associated malignant tumors including NPC.^1^ Moreover NPC is a highly metastatic tumor among all the head and neck cancers.^2^ We have shown that LMP1 alters the gene expression profile of the host cell, and modulates the tumor micro‐environment that upregulates invasion and metastasis, conferring a cancer stem cell‐like property.^3^ LMP1 contains two C‐terminal tail domains containing TES1 (Transformation Effector Site 1, aa187‐231) and TES2 (Transformation Effector Site 2, aa351‐386), that activate signal transduction cascades, including NF‐κB and IRF‐7.^4^

APOBECs proteins are cytidine deaminases that convert cytosine to uracil in host and viral genes. Tumor viral genes such as Human Papillomavirus (HPV) E6/E7 and Human Polyomavirus T antigens induce APOBEC3A (A3A) and APOBEC3B (A3B).^5^, ^6^ Next‐Generation Sequencing analysis has revealed the accumulation of host gene mutations in cancers. NPC exhibits APOBEC signature mutations, accompanying A3A or A3B upregulation.^7^ In addition, EBV infection in gastric and breast cancers is associated with APOBEC3s(A3s) expression and APOBEC signature mutations,^8^, ^9^ implying an unidentified mechanism by which EBV induces A3s expression and host gene mutation. Furthermore, an accumulation of mitochondrial DNA (mtDNA) mutations, such as those found in NPC patients,^10^ reportedly facilitates metastasis via upregulation of reactive oxygen species.^11^

In this study, we sought to determine the mechanism underlying EBV‐mediated mutation via APOBECs, given the close relevance between viral oncogenesis and APOBECs.

## 2 MATERIALS AND METHODS

### 2.1 Data Analysis

For the RNA‐seq of NPC cell lines and the microarray data of LMP1‐TES2‐expressing 293 cells and IRF7‐deficient PBMC, we downloaded the gene expression data from GSE54159^12^, GSE29297^13^, and GSE66486^14^, respectively.

### 2.2 Cell culture, plasmids, transfection

The AdAH cells were maintained in DMEM containing 10% FBS and penicillin/streptomycin. The LMP1 transductant was established as described previously.^3^ The HA‐A3B expression vector was obtained from the NIH AIDS Reagent Program (Cat# 11 090,^15^). FLAG‐GFP, FLAG‐APOBEC3F(A3F), and FLAG‐APOBEC3G(A3G) were constructed as previously described.^16^ The plasmids were transfected into AdAH cells using Effectene (Qiagen, Venlo, The Netherlands), according to the manufacturer's instructions.

As for 293‐EBV cells, the cells were maintained as described in Neuhierl et al^17^ pcDNA LMP1 and LMP1 dominant‐negative form (DN) were generated as described previously.^18^ The plasmids were transfected using TransIT‐LT1 (Mirus, MIR2304), according to the manufacturer's protocol.

The CRISPR activation plasmid for A3B was purchased from Sigma (sc‐401700‐ACT), and transfected with AdAH cells using Lipofectamine2000 (Thermo Fisher), following the manufacturer's protocol. The transfectant was selected using puromycin from the next day of transfection, for 3 days, expanded, and subjected to each analysis.

### 2.3 RT‐PCR

The synthesis of cDNA and qPCR were performed as previously reported.^19^ The primers used are listed in Table S1. The copy numbers of *A3s* and *HPRT1* were determined by absolute quantification, utilizing standard curves, unless otherwise stated. *A2* was relatively quantified by the delta‐delta Ct method, and normalized to *HPRT1*.

### 2.4 Western blotting

Western blotting was performed as described previously.^20^ The antibodies used are listed in Table S2.

### 2.5 APOBEC3H(A3H) genotyping

Total DNA was extracted from the AdAH cells as described previously,^19^ and *A3H* gene corresponding to a.a. pos 15, 18, 121, and 178 was amplified by the primers listed in Table S1, utilizing PrimeStar HS (Takara). The amplicons were sequenced using the primers by which they were amplified.

### 2.6 Clinical samples

Eighteen NPC paraffin‐embedded specimens from the nasopharynx were used for immunohistochemical analysis. The patients were consecutive cases who had been diagnosed at the Division of Pathology and Otolaryngology at Kanazawa University Hospital between January 2005 and March 2012. Specimens obtained at biopsy were from 16 males and 2 females, ages 22‐80 years (mean age 60.1 years), and classified histologically as follows: 14 cases of non‐keratinizing carcinoma (type II) and 4 cases of undifferentiated carcinoma (type III) (5th TNM classification system of the International Union Against Cancer, 1997). There were 4 stage I, 6 stage III, and 8 stage IV specimens. In situ hybridization (ISH) for the detection of EBV‐encoded small RNA (EBER) was performed using the EBER PNA probe/fluorescein and PNA ISH detection kit (Dako, Glostrup, Denmark). All cases were positive for EBER‐ISH. Total DNA was isolated using TaKaRa DEXPAT Easy (Kusatsu, Japan). All samples were collected after obtaining written informed consent from the patients and were used with the approval of the Ethics Committee of Kanazawa University.

### 2.7 Immunohistochemical analyses

Sections were prepared and stained as described previously.^20^ The antibodies used are listed in Table S2. Stained sections were evaluated by two authors (SK and KW), who were independently blinded to the clinical data. The number of immunoreactive cells and that of total cells were counted in three different visual fields, at a magnification of x400. The frequencies of immunoreactive cells were defined as expression scores, and were subjected to statistical analysis.

### 2.8 3D‐PCR analysis

For 3D‐PCR targeting *COI*, *ND2*, and *TP53*, we followed the protocol as described in.^21^ As for *PIK3CA*, the initial PCR was performed as follows: 94°C for 8 min; followed by 40 cycles of 94°C for 1min, 54°C for 30 s, and 72°C for 1min each and a final elongation step at 72°C for 10 min. Nested PCR was performed on a MasterCycler Pro thermal cycler (Eppendorf) as follows: 79.2‐76.6°C for 5 min; followed by 35 cycles of 79.2‐76.6°C for 30 s, 50°C for 30 s, and 72°C for 50 s each and a final elongation step at 72°C for 10 min. To identify mutations, GenBank accession number NC_012920.1 was used as a reference sequence for human *COI*. The primers used were listed in Table S1.

### 2.9 Colony assay

We utilized the CytoSelect 96‐well Cell Transformation Assay Kit (Cell Biolabs, CBA‐135), according to the manufacturer's protocol. Briefly, 150 cells per a well of a 96 well plate were suspended in agar matrix, and loaded on a basal agar matrix. The cells were incubated for 14 days, and subjected to MTT assay. The cell numbers were quantified by measuring the absorbance at 570 nm.

### 2.10 Migration assay

The assay was performed by utilizing Corning BioCoat Matrigel Invasion Chambers with an 8.0 µm PET Membrane (354 480), according to the manufacturer's protocol. Briefly, the cells were suspended in DMEM not containing FBS, and 125 000 cells per an insert for a 24‐well plate, were loaded on an insert. The companion 24‐well plate was filled with DMEM containing 10% FBS, and after 24‐hour incubation, the remaining cells in the insert were wiped out, and the migrated cells were stained using Diff‐Quick (Sysmex, 16 920) and counted.

### 2.11 Statistical analysis

Statistical analyses were performed using GraphPad Prism (GraphPad Software, La Jolla, California) or IBM SPSS Statistics version 23 (IBM, Armonk, NY). Two‐tailed unpaired t‐tests were used for RT‐qPCR analysis and IHC scoring, and Pearson's chi‐squared tests were used for mutation analyses. For the correlation of IHC scoring, Pearson's correlation test was used. Differences between experimental groups with *P* values < .05 were considered statistically significant.

## 3 RESULTS

### 3.1 EBV‐LMP1 alters A3s expression profile

To verify an association between EBV infection and A3s expression, we checked the available databases for the expression level of A3s in EBV (+) and (‐) NPC cell lines. From GSE54159 containing the RNA‐seq result of four nasopharyngeal cell lines,^12^ we found that the expression level of *A3B, A3F, and A3H* was higher in EBV(+) cells C666 and X666, compared to the EBV(‐) cells HK1 and NP460 (Figure S1, the data for other *A3s* were unavailable).

**Figure 1:**
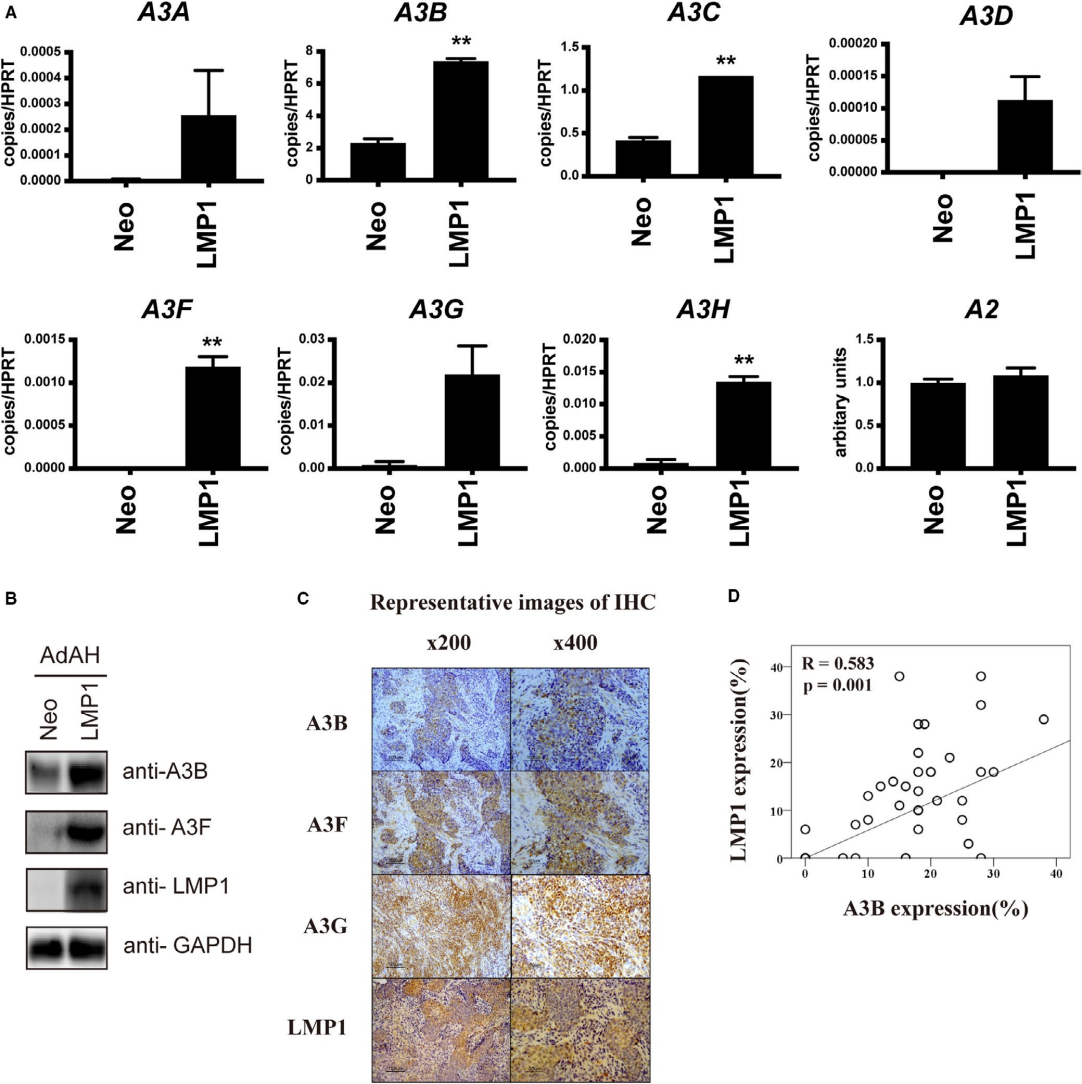
APOBEC expression in LMP1‐expressing cells and NPC specimens (A) RT‐qPCR analysis of AdAH cells retrovirally transduced with LMP1, along with the neomycin‐resistance gene. As for qPCR, the samples were duplicated and the results were normalized to *HPRT*. The levels of *A3s* are indicated as copy numbers per *HPRT*, and that of *A2*, as arbitrary units (error bars; SEM, **P < .01). (B) Total cell lysates of each transductant were subjected to SDS‐PAGE, and immunoblotted with antibodies against A3B, A3F, LMP1, and GAPDH. (C) Representative analysis of Immunohistochemical (IHC) analysis in NPC specimens. The sections were stained with antibodies against LMP1, A3B, A3F, and A3G. (D) The frequency of A3B or LMP1 expressing cells was calculated. P values were calculated using unpaired two‐tailed Student's t‐test and Pearson's correlation test for the RT‐qPCR analysis and IHC scoring, respectively

Furthermore, to clarify whether LMP1 induces A3s, an EBV(‐) nasopharyngeal cell line, AdAH, was retrovirally transduced with EBV‐LMP1, and transcription of A3s in LMP1‐expressing cells was evaluated by RT‐qPCR (Table S1). The analysis revealed that, transcripts of *A3B*, *APOBEC3C(A3C)*, *A3F*, and *A3H*, were significantly upregulated detected in the LMP1 transductant, compared to the control (neomycin‐resistance gene transductant, Figure 1A). Those of *A3A*, *APOBEC3D(A3D)*, and *A3G* were also increased, although the difference was not statistically significant between the two cell lines. *APOBEC2* was altered within a 1.09‐fold difference. Western blot analysis was further performed(Table S2), and revealed that the protein level of A3B and A3F was consistently increased in the LMP1 transductant, compared to the Neo control (Figure 1B). A3C and A3H were undetectable (data not shown). Since the A3H haplotype reportedly affects protein stability, we sequenced the A3H gene of AdAH cells and found that it was homologous to hap III (Figure S2).

To further verify the correlation between the protein level of A3s and LMP1 in vivo, we performed immunohistochemical analysis of these proteins in biopsy sections of NPC specimens (Table S2 and Figure 1C). We found a significant correlation of LMP1 protein expression with A3B (n = 39, R = 0.583, *P* = .001), but not with A3F or A3G (Figure 1D). Taken together, these results suggest that EBV‐LMP1 alters the expression profile of A3s in nasopharyngeal cells.

LMP1 reportedly activates various intracellular signaling, including IRF7 and NF‐kB.^4^ By mining the public microarray data GSE66486^14^, we found that IRF7‐deficient PBMC expresses lower levels of A3F and A3G, but comparable levels of A3B, than the control, when challenged with influenza A virus (Figure S3). We attempted, but failed to establish IRF‐7‐deficient LMP1 expressing AdAH cells (data not shown), implying its essential role in survival. In addition, we mined another data, GSE29297, where the expression of the TES1‐dead LMP1 mutant was induced by addition of tetracycline.^13^ We found that its expression induced the expression of A3B, which was attenuated when IkB super‐repressor (the N‐terminus deletion mutant, resistant to IKK‐mediated degradation) was co‐expressed (Figure S4). Taken together, these findings suggest that LMP1 induced A3s expression, and TES2 might be responsible for A3B induction, dependent on the NF‐kB signaling pathway.

### 3.2 LMP1 and A3s hypermutate mtDNA

**Figure 2:**
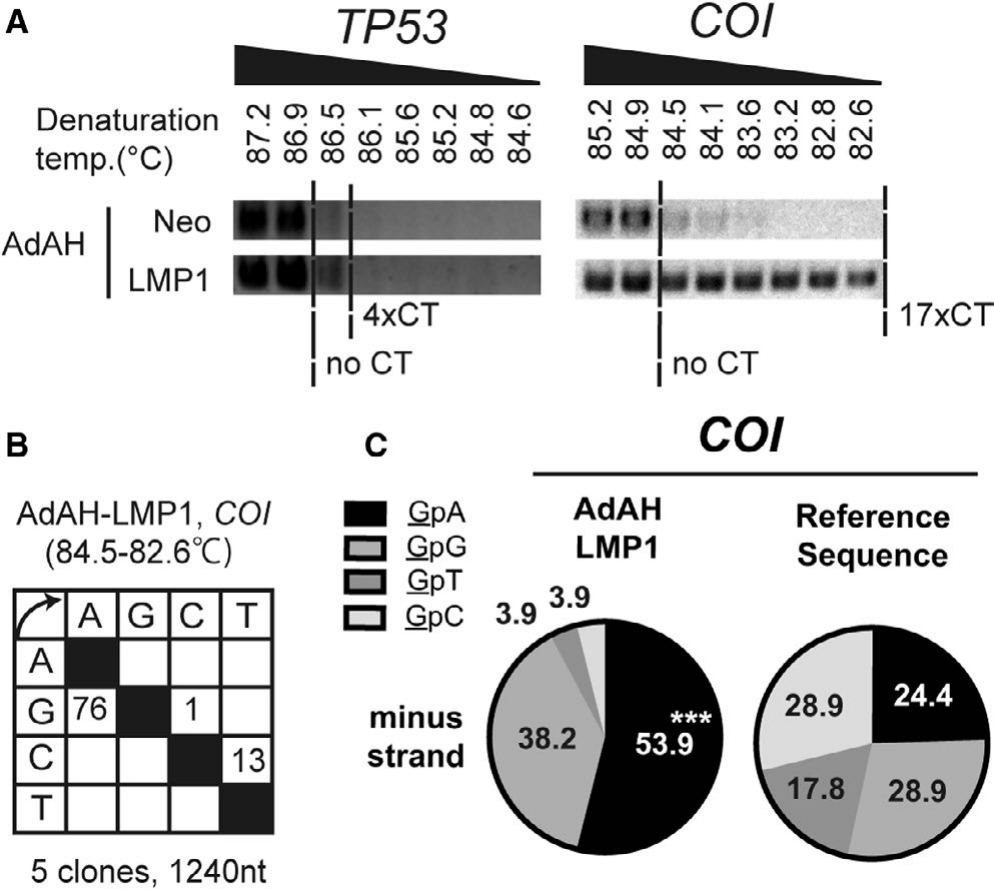
LMP1 hypermutates mtDNA (A) AdAH cells retrovirally transduced with LMP1 were subjected to 3D‐PCR analysis targeting *TP53* or *COI*. No CT, 4xCT, and 17xCT indicate the lowest denaturation temperature at which the target sequence is amplified from the control plasmids containing 0, 4, and 17 C‐to‐T mutations, respectively. (B,C) The *COI* amplicons from a denaturing temperature of 84.5°C or lower were sequenced (B), and the dinucleotide preference of G‐to‐A mutations are summarized (C). P‐values were calculated using chi‐square tests for the dinucleotide analysis. ***P < .005

To clarify the consequence of A3s induction by LMP1, we performed 3D (Differential DNA Denaturation)‐PCR, a highly sensitive method to quantify GC to AT substitution, with low denaturing temperature^21^ (Table S1). Total DNA from LMP1 or Neo transductants was subjected to 3D‐PCR analysis targeting *TP53*, one of the nuclear genes significantly mutated in NPC,^22^ and the mitochondrial gene *COI*, following the protocol in.^21^ We found that while *TP53* was amplified with the denaturation temperature of 86.5°C or higher, comparable to 4 × CT (positive control, ^19^) in each cell. In contrast, *COI* was amplified from the LMP1, but not Neo transductant, with 82.6°C, the lowest at which it is amplified from 17 × CT^19^ (Figure 2A). Sequencing the amplicon from lower denaturation temperature than 84.9°C (the lowest temperature at which no mutation control (no CT) is amplified), revealed accumulation of G‐to‐A biased mutations (Figure 2B). The dinucleotide context of deaminated cytosines was biased toward TpC (Figure 2C), consistent with the one induced by A3B.^5^ We also performed another 3D‐PCR targeting nuclear gene *PIK3CA* and mitochondrial gene *ND2*, whose 3D‐PCR protocol was available.^21^We found that hypermutation of *ND2,* but not that of *PIK3CA,* was increased (Figure S5).

**Figure 3:**
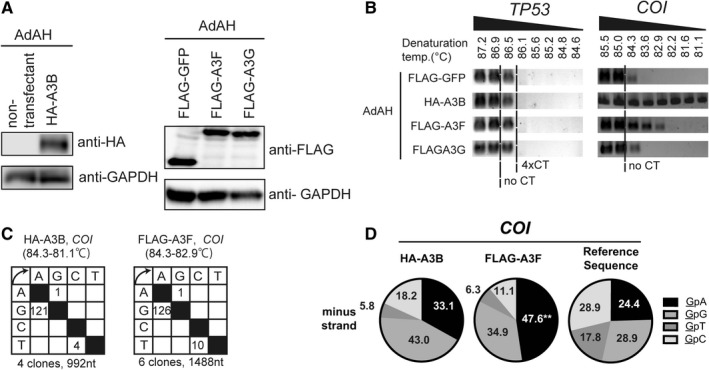
A3B and A3F hypermutate mtDNA (A,B) AdAH cells were transfected with expression vectors for HA‐A3B, FLAG‐GFP, FLAG‐A3F, and FLAG‐A3G. Two days after transfection, the cells were harvested and used for immunoblotting (A) and 3D‐PCR analysis (B) as in Figure 2. No CT and 4xCT indicate the lowest denaturation temperature at which the target sequence is amplified from the control plasmids containing 0 and 4 C‐to‐T mutations, respectively. (C,D) The *COI* amplicons from a denaturing temperature of 84.3°C or lower were sequenced (C), and the dinucleotide preference of G‐to‐A mutations are summarized (D). P‐values were calculated using chi‐square tests for the dinucleotide analysis. **P < .01

Further to verify whether expression of A3s is sufficient to hypermutate mtDNA, FLAG‐ or HA‐tagged A3s were overexpressed and 3D‐PCR analysis was performed. The result revealed that *COI* was amplified from the A3B and A3F, but not from the GFP or A3G transfectants, with lower denaturation temperature than 84.3°C (Figures 3A and 3B). As for *TP53*, it was amplified from none of them, with 86.1°C or lower. Sequencing the *COI* amplicons from lower denaturation temperature also revealed G‐to‐A biased mutations, as found from the LMP1 transductant (Figure 3C). Dinucleotide analysis revealed a bias toward TpC or CpC (Figure 3D). These results suggested that LMP1 and A3s mutate the mitochondrial genome.

Further to verify whether *COI* hypermutation occurs in vivo, total DNA from NPC specimens was subjected to 3D‐PCR. We found that *COI* was amplified from 9 out of 18 samples, with the denaturing temperature of 84.1°C or lower (more than 4xCT, Figure S6A). *TP53* hypermutation was comparable to 4xCT, in 5 samples where *COI* was hypermutated. Sequencing analysis revealed G‐to‐A bias and TpC or CpC preference (Figures. S6B and S6C), as in the LMP1 transductant. The result suggested that mtDNA was hypermutated in the nasopharyngeal tumor specimens, consistent with the in vitro experiments.

To further verify a correlation between EBV infection and *COI* hypermutation, we set the denaturation threshold at 84.1°C (4 × CT), and sorted the cases into those positive or negative for hypermutation. We found that hypermutation was detected in 4 out of 6 *LMP1*(+), and 5 out of 12 *LMP1*(‐) cases (Figure S6D) In addition, it was found in 9 out of 14 cases histologically diagnosed with type II carcinoma (non‐keratinizing), but none of 4 cases with type III carcinoma (undifferentiated).

We further overexpressed LMP1 in the 293 producer cell line where upon induction the EBV genome can replicate.^17^ We found that LMP1 increased A3B both at the mRNA and protein level, and hypermutated *COI* (Figure S7). The LMP1‐DN, both of whose TES1 and TES2 contain mutations,^18^ upregulated A3B to a lesser extent than the wildtype, but did not hypermutate *COI*. Taken together, the results suggested that LMP1 mutates the mitochondrial DNA, possibly dependent on TES domains.

### 3.3 Expression of LMP1 and A3B is associated with neck metastasis of NPC

**Figure 4:**
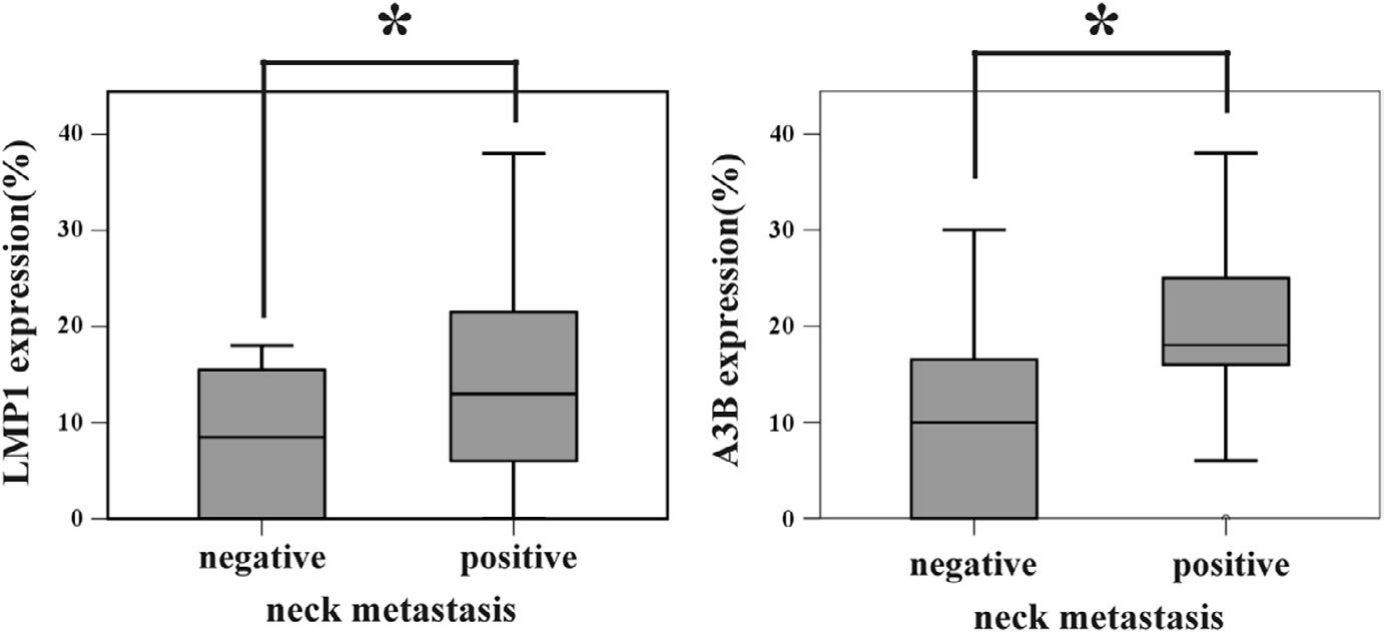
Expression of LMP1 and A3B proteins positively correlates with neck lymph node metastasis Nasopharyngeal cancer specimens were subjected to immunohistochemical analysis to quantify the protein level of LMP1, A3B, A3F, and A3G, as in Figures 1C and 1D. The expression scores of patients with or without neck metastasis are summarized. P values were calculated using the unpaired two‐tailed Student's t‐test. (error bars; SD,* *P* < .05)

To determine the significance of LMP1‐mediated A3s induction in NPC, the correlation between neck metastasis and the expression of A3s proteins was examined, as mtDNA mutation is reportedly associated with distant metastases.^11^ As in Figure 4, the IHC scores of LMP1 and A3B, were higher in cases with neck metastasis, compared to those without metastasis (LMP1: metastasis (‐): 7.9 ± 7.66, metastasis (+): 14.7 ± 11.7, *P* = .04, A3B: metastasis (‐): 9.8 ± 10.1, metastasis (+): 19.0 ± 8.1, *P* = .01). Those of A3F or A3G were comparable between the two groups (A3F; metastasis (‐): 21.9 ± 12.68, metastasis (+): 18.48 ± 11.14, *P* = .42, A3G; metastasis (‐): 17.9 ± 10.5, metastasis (+): 14.0 ± 10.7, *P* = .29). This result implied involvement of A3B in neck metastasis.

Furthermore, to verify the consequence in vitro, endogenous A3B expression was activated by transfecting a CRISPR‐activation vector (Figures. S8A‐C), and the transfectants were subjected to colony formation assay and migration assay (Figures S8D and S8E). However, the ability of colony formation, as well as that of migration, was comparable with its parental cells, suggesting that overexpression of A3B is not sufficient to acquire cancer‐related phenotype in vitro.

## 4 DISCUSSION

In this study, using LMP1‐expressing cells and NPC specimens, we demonstrated that EBV‐LMP1 induces A3B and A3F (Figure 1). Expression of LMP1, A3B, and A3F resulted in hypermutation of mtDNA (Figures 2 and 3), and the protein level of LMP1 and A3B was found to be correlated with neck metastasis in NPC (Figure 4). This study is the first to demonstrate that EBV gene expression leads to A3B/A3F expression, and mtDNA mutation.

LMP1 reportedly activates many intracellular signaling pathways, including the IRF7 and NF‐kB pathways.^4^ We found that A3F and A3G were positively regulated by IRF‐7 in the PBMC challenged with influenza A virus (Figure S3), while A3B is induced by LMP1‐TES2, in an NF‐kB dependent manner (Figure S4). Consistent with this, NF‐kB reportedly mediates PMA‐mediated A3B induction, in a mammary epithelial cell line, MCF10A.^23^ It is intriguing to speculate, yet remains to be verified in detail, that A3B and A3F/G are differentially induced by NF‐kB and IRF7, respectively.

NPC reportedly contains APOBEC signature mutations that associate with A3A or A3B upregulation.^7^ Additionally, in gastric and mammary tumors, EBV infection is associated with A3s expression and APOBEC signature mutation.^8^, ^9^ Consistently, we found that EBV‐LMP1 increased A3s expression, identifying a viral gene responsible for A3s upregulation (Figure 1). Nonetheless, our 3D‐PCR analysis did not detect increased mutation of nuclear genes, *TP53* and *PIK3CA*, by A3s or LMP1 either in vitro or in vivo (Figures 2A and 3B, and Figs. S5 and S6A). It remains to be elucidated, whether A3s require other factors to hypermutate nuclear genes, or they evade from A3s by unknown mechanisms.

In AdAH cells, LMP1 significantly increased transcription of *A3C* and *A3H* (Figure 1A) but did not increase the protein level (Figure 1B). We cannot exclude possible issues such as the low sensitivity of the antibodies used for Western blot analysis, the inefficient translation of the mRNA, or the relative stability in this cell line of A3C or A3H compared to that of A3B or A3F. Intriguingly, the haplotypes of A3H reportedly affect its stability^24^ and A3H is homologous to hap III (Figure S2), which is associated with low stability, possibly explaining the discrepancy. Further studies are necessary to clarify the mechanism that regulates A3 protein stability.

The A3B protein level in NPC was correlated with neck metastasis (Figure 4). In metastatic lymph nodes of breast cancer, A3A and A3B are expressed more abundantly and their mutation signature is found more frequently, compared to that in the primary lesions.^25^ As A3s expression is believed to promote metastasis and other events related to cancer evolution,^26^ and mtDNA mutation experimentally facilitates metastasis via ROS upregulation,^11^ it is intriguing to speculate that LMP1 facilitates metastasis, at least in part, by hypermutating mtDNA (Figure S9). Nonetheless, A3B overexpression did not increase colony formation or migration in vitro (Figures S8D and S8E). We speculate that it requires long‐term expression of A3B for the cells to accumulate mutation and to acquire metastatic phenotype. And in addition to A3B, A3F hypermutated mtDNA in vitro (Figure 3B), and the other groups have reported that A3G promoted liver metastasis of colon cancer in a murine model, possibly by altering microRNA (miRNA) expression.^27^ Thus, we do not deny the possibility that A3s other than A3B might contribute to metastasis, either by mutating the host (both nuclear and mitochondrial) DNA, changing the miRNA profile, or other unknown mechanisms. In addition, we have reported other factors involved in LMP1‐mediated metastasis, such as RAGE.^28^ Thus, the extent that mtDNA mutation contributes to LMP‐1‐mediated metastasis remains yet to be determined.

We found a higher frequency of *COI* hypermutation in *LMP1* mRNA(+) cases compared to those in *LMP1*(‐), although this finding lacked statistical significance, possibly due to the limited number of cases. *COI* hypermutation was significantly associated with histological type II carcinoma (non‐keratinizing) (Figure S6D). Given that type II is more differentiated that type III,^2^ this corroborates our findings that keratinocyte differentiation induces mtDNA hypermutation.^19^ Thus, it is fair to speculate that both LMP1 expression and host cell differentiation contribute to mtDNA hypermutation in vivo. However, the detailed mechanism of mtDNA hypermutation involving A3s and host cell differentiation remains yet to be determined.

A3s were originally discovered as antiviral molecules that destroy viral genomic information, especially that of retroviruses such as human immunodeficiency virus.^29^ As for EBV, Suspene et al reported that viral genes *EBNA1* and *EBNA2* are hypermutated in UNG‐ or AID‐deficient immortalized B lymphocytes,^30^ raising the possibility that A3s may suppress EBV propagation via editing of the viral genome. However, Cheng et al reported that another viral gene, BORF2, protects the viral genome from A3B‐mediated hypermutation in EBV‐replicating gastric cells,^31^ demonstrating that replicating viral genome is protected from, rather than mutated by, A3s. Thus, A3s are unlikely to counteract propagation of wild type EBV, although inhibiting BORF2 may enable A3s to hypermutate viral genome and consequently prevent viral replication. Whether these findings hold true in nasopharyngeal cells remains yet to be verified.

In summary, we demonstrated that EBV‐LMP1 induces A3B/A3F expression and mutates mtDNA. Further studies are necessary to completely understand the molecular mechanism by which A3s are induced by viral genes, the landscape of mutations created by viral genes, and the consequence of host genome mutation by the LMP1‐A3s axis.

## CONFLICT OF INTEREST

The authors declare no conflicts of interests.

## AUTHOR CONTRIBUTIONS

K Wakae, S Kondo, M Muramatsu, and T Yoshizaki contributed to study conception and design. K Wakae, S Kondo, HT Pham, L Que, Y Li, X Zheng, K Fukano, and MM Kita contributed to data acquisition. K Wakae, S Kondo, K Kitamura, KWatashi, H Aizaki, M Muramatsu, and T Yoshizaki contributed to data analysis and interpretation. S Kondo, N Wakisaka, T Ueno, K Ishikawa, Y Nakanishi, and K Endo contributed to material supports. K Wakae, S Kondo, M Muramatsu, and T Yoshizaki contributed to manuscript preparation.

## Supporting information

Supplementary Material

## Data Availability

The data that support the findings of this study are available from the corresponding author upon reasonable request.
