# Supplementary material for: EBV‐LMP1 induces APOBEC3s and mitochondrial DNA hypermutation in nasopharyngeal cancer
Source: Cancer Med. 2020 Aug 20;9(20):7663–71. doi: 10.1002/cam4.3357 (PMC7571841; doi:10.1002/cam4.3357)
Supplement: Supplementary file 1 — Supplementary Material [file CAM4-9-7663-s001.pdf]

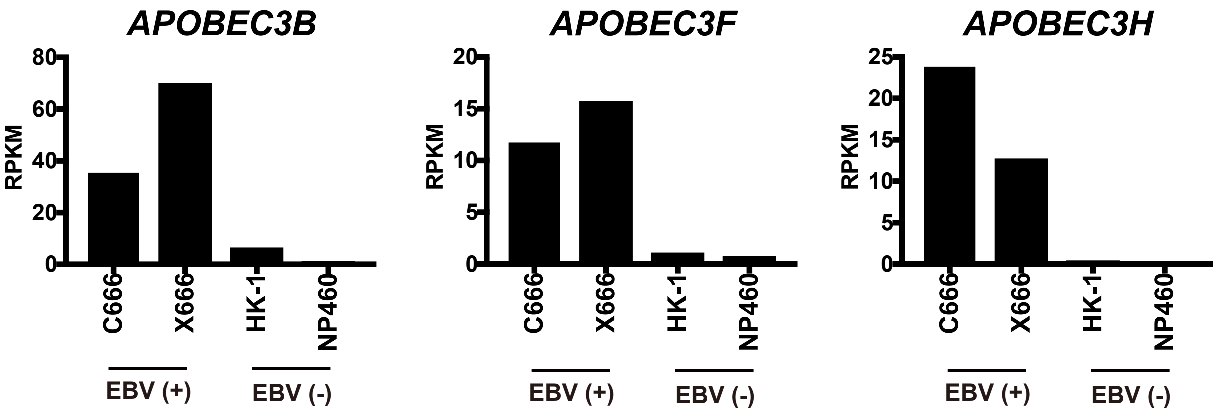

Supplementary Figure S1. ***A3s*** **are upregulated in EBV(+) NPC cell lines**
RNA-seq data was downloaded from GSE54159 (1), and the RPKM values of *APOBEC3B*, *3F*, *3H*, were extracted.

(1)Szeto CY, Lin CH, Choi SC, et al. Integrated mRNA and microRNA transcriptome sequencing characterizes sequence variants and mRNA-microRNA regulatory network in nasopharyngeal carcinoma model systems. FEBS Open Bio 2014; 4: 128-140.

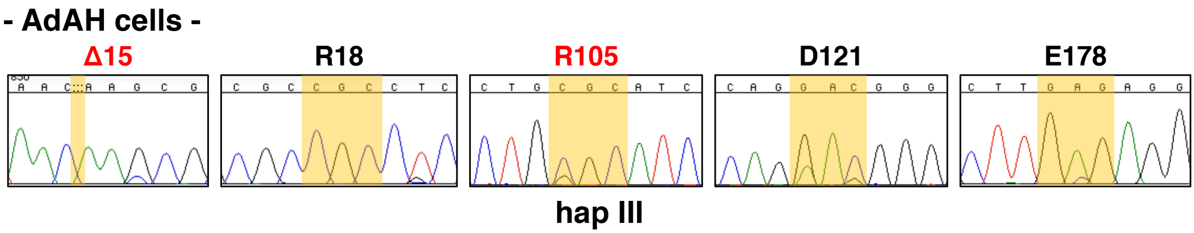

Supplementary Figure S2. A3H haplotype of AdAH cells
The sequence of A3H gene, corresponding to amino acid residue 15, 18, 105, 121, and 178 are indicated.

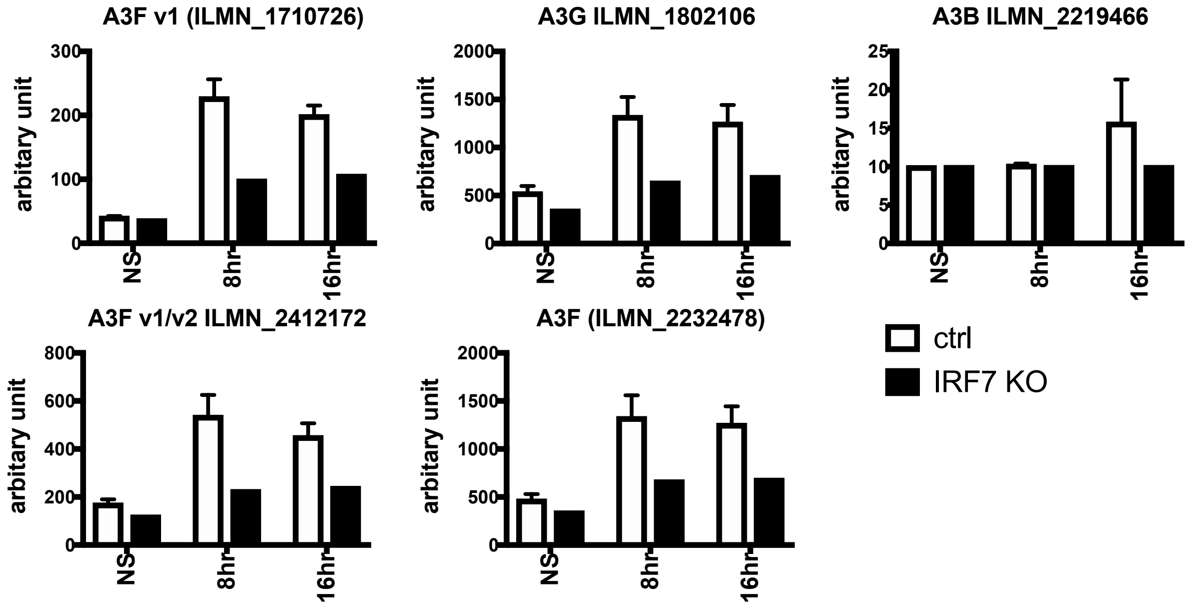

Supplementary Figure S3.
A3s expression in influenza A virus-challenged, IRF7-deficient PBMC

Expression values of APOBEC3F, 3G, and 3B were extracted from GSE66486 (2).

(2) Ciancanelli MJ, Huang SX, Luthra P, et al. Infectious disease. Life-threatening influenza and impaired interferon amplification in human IRF7 deficiency. Science. 2015; 348: 448-53.

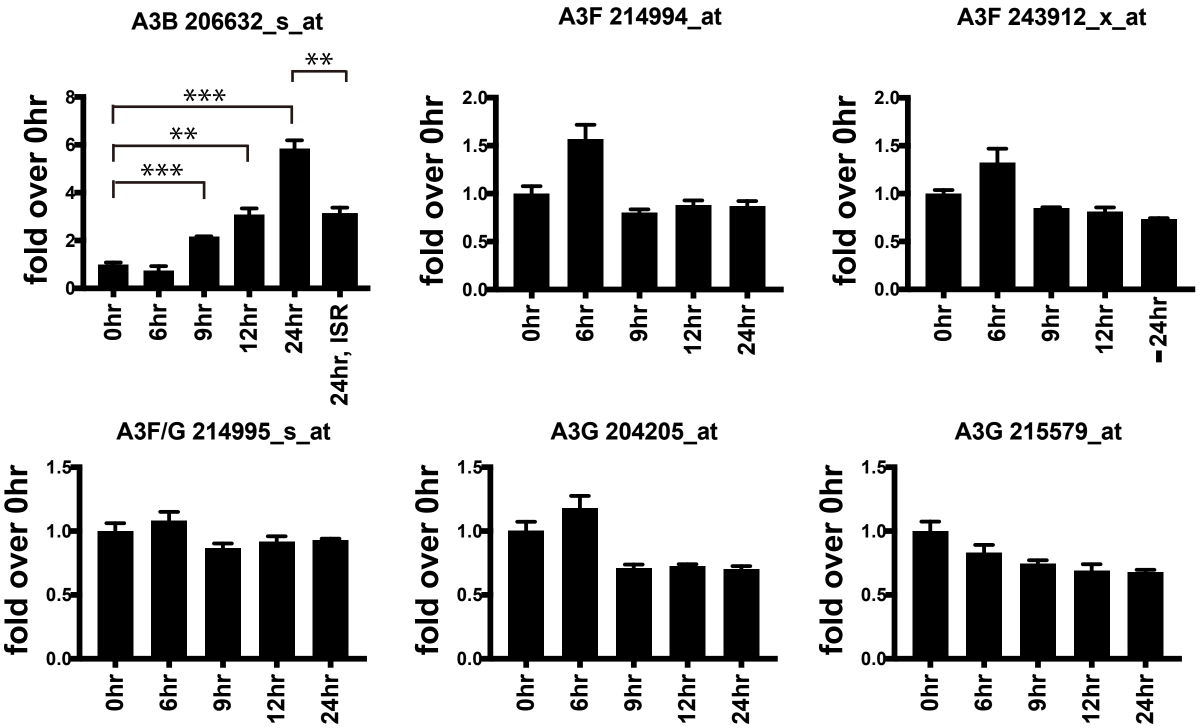

Supplementary Figure S4.
TES2 domain of LMP1 induces A3B expression, via NF-kB

Microarray data was downloaded from GSE29297 (3), and the expression levels of APOBEC3B, 3F, and 3G were extracted. The result is shown by the fold induction over 0hr.

(3)Gewurz BE, Mar JC, Padi M, et al. Canonical NF-kappaB activation is essential for Epstein-Barr virus latent membrane protein 1 TES2/CTAR2 gene regulation. J Virol. 2011; 85: 6764-73.

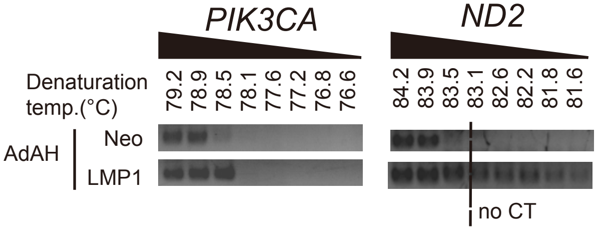

Supplementary Figure S5. 3D-PCR analaysis of AdAH-LMP1 transductant for a nuclear gene, PIK3CA, and a mitochondrial ND2.

Total DNA from AdAH cells retrovirally transduced with neomycin resistant genes or LMP1, were subjected to 3D-PCR analysis targeting *PIK3CA* or *ND2*, as in Fig.2. No CT indicates the lowest denaturation temperature at which the target sequence is amplified from the control plasmid containing no C-to-T mutation (4).

(4) Wakae K, Nishiyama T, Kondo S, et al. Keratinocyte differentiation induces APOBEC3A, 3B, and mitochondrial DNA hypermutation. Sci Rep. 2018; 8: 9745.

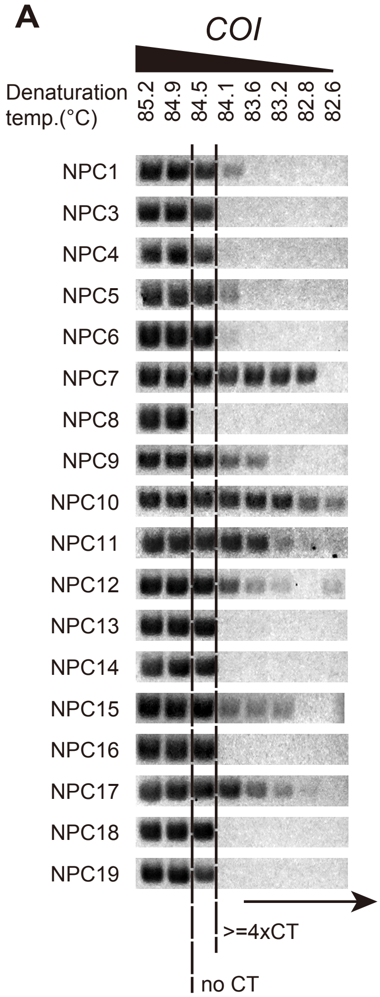

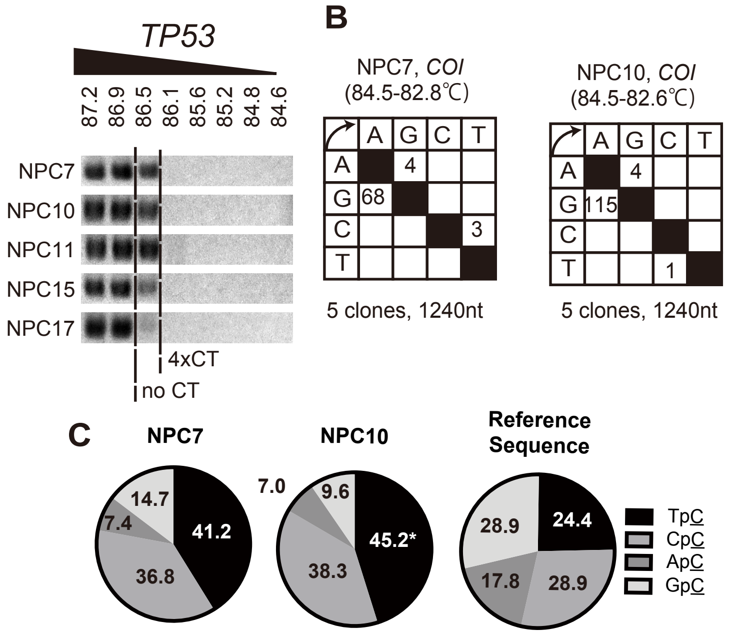

Supplementary Figure S6.
3D-PCR analysis of NPC specimens

(A)Total DNA from NPC tissues was subjected to 3D-PCR analysis targeting a host gene *TP53*, and a mitochondrial gene *COI*. For reference, the lowest temperature at which the gene was amplified from each control plasmid was indicated as dot lines (2). (B, C) The *COI* amplicons from a denaturing temperature of 84.5 °C or lower were sequenced (B), and the dinucleotide preference of G-to-A mutations was summarized (C). (D) Correlation between LMP1, histology, and *COI* hypermutation. (*P<0.05)

D

LMP1		mtDNA hypermutation (<=84.1° C)	% of hypermutation
+	NPC1	+	66.7%(4/6)
	NPC4	−	
	NPC5	+	
	NPC7	+	
	NPC17	+	
	NPC19	−	
−	NPC3	−	41.7%(5/12)
	NPC6	−	
	NPC8	−	
	NPC9	+	
	NPC10	+	
	NPC11	+	
	NPC12	+	
	NPC13	−	
	NPC14	−	
	NPC15	+	
	NPC16	−	
	NPC18	−	

Histology		mtDNA hypermutation (<=84.1° C)	% of hypermutation
non-keratinizing carcinoma (type II)	NPC1	+	64.2%(9/14)
	NPC3	−	
	NPC5	+	
	NPC6	−	
	NPC7	+	
	NPC8	−	
	NPC9	+	
	NPC10	+	
	NPC11	+	
	NPC12	+	
	NPC13	−	
	NPC15	+	
	NPC17	+	
	NPC19	−	
undifferentiated carcinoma (type III)	NPC14	−	0%(0/4)
	NPC16	−	
	NPC18	−	
	NPC4	−	

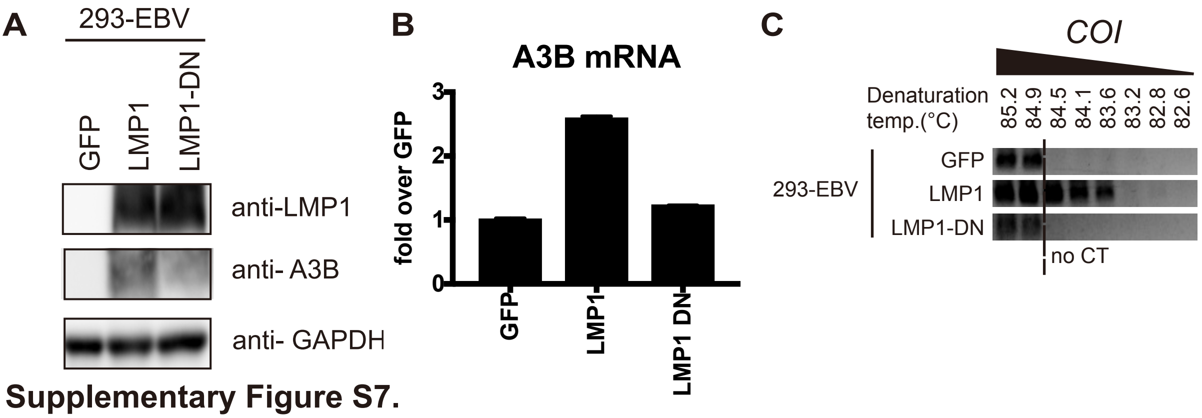

Supplementary Figure S7.

LMP1 induces A3B expression in an EBV-replicating 293 cells

293-EBV cells (5) were transfected with pcDNA wildtype or dominant-negative(DN) LMP1, and were subjected to (A) Western blotting analysis,(B) RT-qPCR analysis, and (C) 3D-PCR analysis targeting COI. As for RT-qPCR, the mRNA level was quantified by the 2ˆ(–delta delta CT) method, and normalized by HPRT.

(5)Delecluse HJ, Hilsendegen T, Pich D, Zeidler R, Hammerschmidt W. Propagation and recovery of intact, infectious Epstein-Barr virus from prokaryotic to human cells. Proc Natl Acad Sci U S A. 1998; 95: 8245-50.

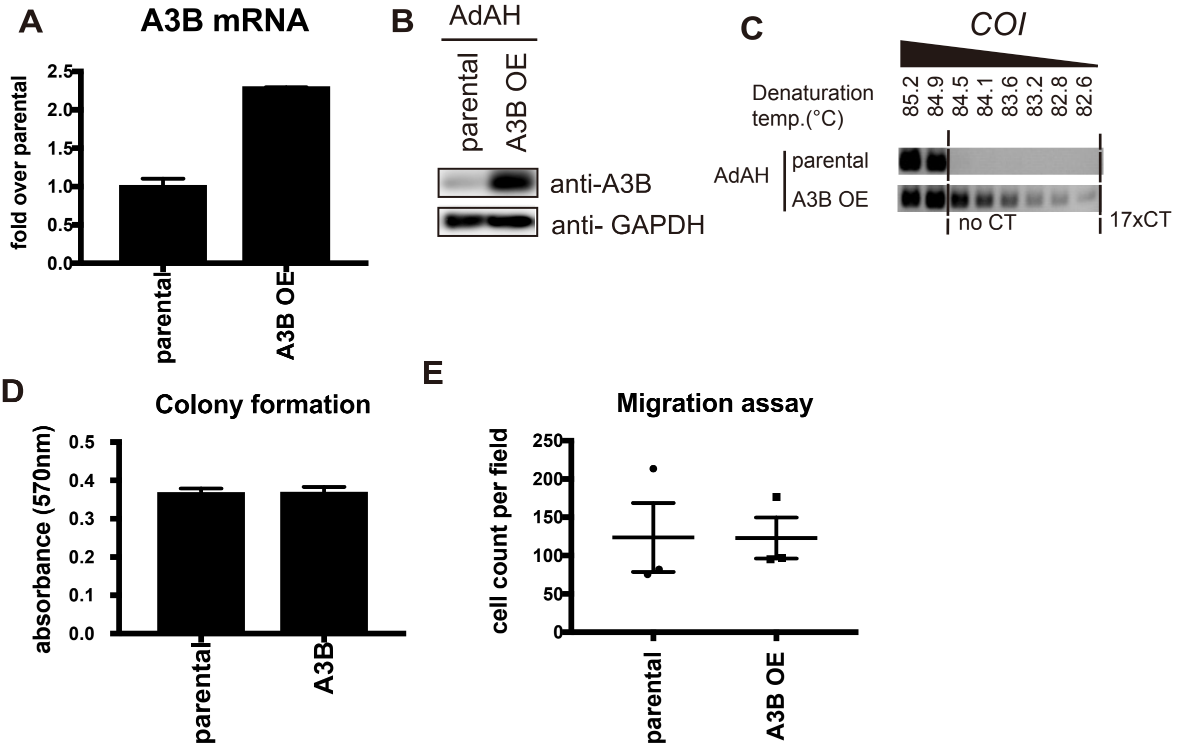

Supplementary Figure S8.

Colony formation assay and migration assay of A3B overexpressing AdAH cells

AdAH cells were transfected with a CRISPR activation plasmid for A3B, (A,B) A3B overexpression was validated by RT-qPCR (A) and Western blotting analysis (B). (C) 3D-PCR analysis targeting COI. (D, E) Colony assay and migration assay. As for RT-qPCR, the mRNA level was quantified by the 2ˆ(–delta delta CT) method, and normalized by HPRT.

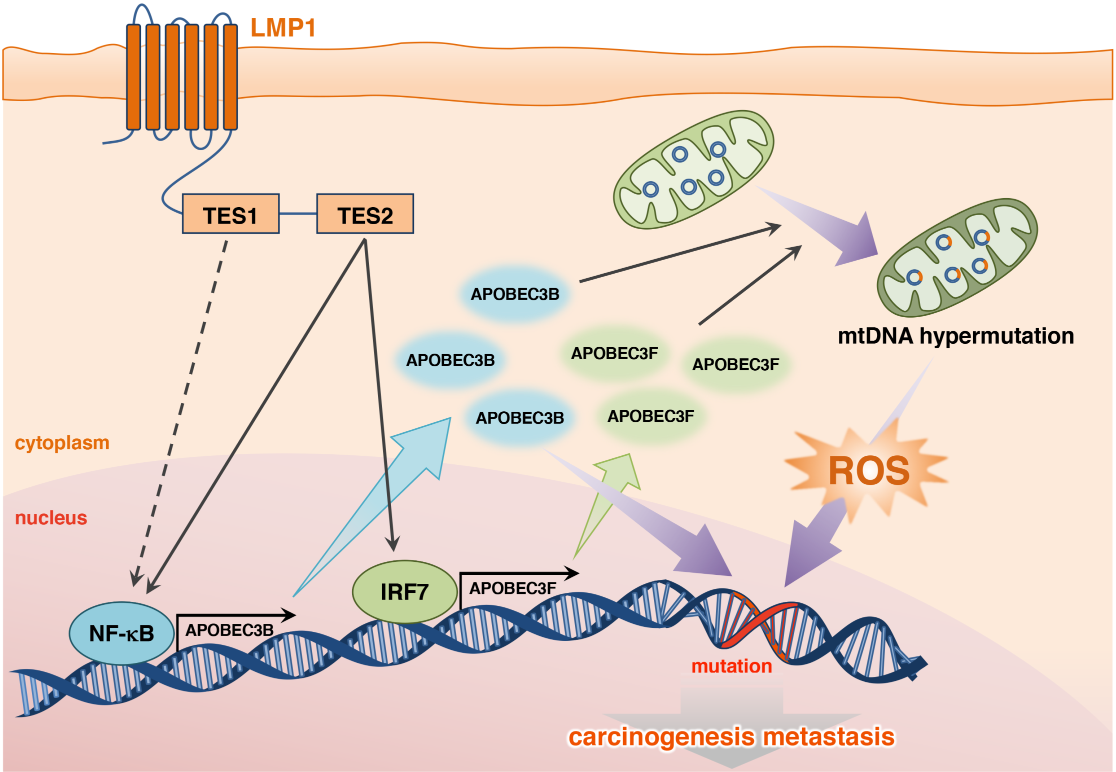

Supplementary Figure S9.
The hypothesis of accumulating host gene mutation, induced by LMP1-APOBEC axis.

Supplementary Table S1: Primers used in this study

assay	Figures	target genes	orientation	sequence	reference
3D-PCR	Figs. 2 and 3, and Supplementary Fig. S3	*COI*, outer	F	GCGGTTGACTATTCTCTACAAACCACAAA	Suspene et al. Proc. Natl. Acad. Sci. 2011<br>Wakae et al. Sci. Rep.2018
			R	GGGGGTTTTATATTGATAATTGTTGTGATGAAA	
		COI, inner	F	CGTTATCGTCACAGCCCATGCATTTGTAA	
			R	GAGGAGACACCTGCTAGGTGTAAGGTGAA	
		ND2, outer	F	ATAGCAGTTCTACCGTACAACCCTAA	
			R	GGGAGATAGGTAGGAGTAGCGTGGTAA	
		ND2, inner	F	ACCGCATTCCTACTACTCAACTTAAA	
			R	GGTGGAGTAGATTAGGCGTAGGTAGAA	
		TP53, outer	F	GAGCTGGACCTTAGGCTCCAGAAAGGACAA	
			R	GCTGGTGTTGTTGGGCAGTGCTAGGAA	
		TP53, inner	F	TTCTCTTTTCCTATCCTGAGTAGTGGTAA	
			R	AAAGGTGATAAAAGTGAATCTGAGGCATAA	
		PIK3CA, outer	F	GGTTCTTTCCTGTCTCTGAA	this study
			R	GTGCCAACTACCAATGTAGTA	
		PIK3CA, inner	F	GCTTTTTCTGTAAATCATCTGTG	
			R	GCTGAGATCAGCCAAATTCAGTT	
RT-qPCR	Fig.1, Fig.S4	*A3A*	F	ATGGCATTGGAAGGCATAAG	Liang et al. Proc. Natl. Acad. Sci. 2013<br>Kitamura et al. Plos Pathogens 2013<br>Wang et al. J. Virol 2014<br>Wakae et al. Virology 2015<br>Wakae et al. Sci. Rep.2018
			R	CAAAGAAGGAACCAGGTCCA	
		A3B	F	TTCGAGGCCAGGTGTATTTCA	
			R	CAGAGATGGTCAGGGTGACA	
		A3C	F	CAACGATCGGAACGAAACTT	
			R	TATGTCGTCGCAGAACCAAG	
		A3D	F	ACCCAAACGTCAGTCGAATC	
			R	GCTCAGCCAAGAATTTGGTC	
		A3F	F	GAAACACAGTGGAGCGAATG	
			R	GAAATGGGGCTCTGATGAAAG	
		A3G	F	GGTCAGAGGACGGCATGAGA	
			R	GCAGGACCCAGGTGTCATTG	
		A3H	F	CCCGCCTGTACTACCACTGG	
			R	GGGTTGAAGGAAAGCGGTTT	
		HPRT1	F	GCCCTGGCGTCGTGATTAGT	
			R	CGAGCAAGACGTTCAGTCCTGTC	
		A2	F	CCAGGCTGCTCTGAAGAAGC	this study
			R	AGGCCTTGGATTCACCCTCT	
		LMP1	F	CCCTTTGTATACTCCTACTGATGATCAC	Kubota et al. J Virol Methods. 2008
			R	ACCCGAAGATGAACAGCACAAT	
A3H haplotyping	Supplementary Fig.S2	aa15 and 18	F	GGTGACTCAAGAGGACGCTCCCTTCATCTTT	this study
			R	CTGTGCCCAGAGAGCCCGTGTGG	
		aa105 and 121	F	AGTCACATGACTCCTGGCCTCTCTC	
			R	TGGGAAGCCCATGACCTCCACCGG	
		aa178	F	CTCCTGGCACTGCAGCTGCTGCCC	
			R	GCAGTGGGCAGAGGTGTGACTGGGTATG	

Supplementary Table S2: Antibodies used in this study

assay	Figures	target	origin	reference
WB	Figs.1 and 3	A3B	serum from a rabbit immunized with A3AB peptide, CDEHSQALSGRLRAILQNQGN	Kondo et al. Oncogene 2017<br>Wakae et al. Sci Rep. 2018
		A3D	sigma, HPA055116	Seishima et al. Sci. Rep. 2018
		A3F	serum from a rabbit immunized with A3F peptide, CFVYNDDEPFKPWKGLKYN	
		A3G	serum from a rabbit immunized with A3G peptide, CQDLSGRLRAILQNQEN	Kitamura et al. Plos Pathogens 2013<br>Wang et al. J. Virol. 2014<br>Seishima et al. Sci. Rep. 2018
		A3H	sigma,HPA021492	Seishima et al. Sci. Rep. 2018
		LMP1	abcam, ab78113	
		HA	invivogen, #ab-hatag	
		FLAG	Sigma, F3165	
		GAPDH	Sigma Aldrich, G9545	
IHC	Figs.1 and 4	LMP1	Dako, M0897	
		A3B	Abcam, ab184990	Kondo et al. Oncogene 2017<br>Seishima et al. Sci. Rep. 2018
		A3F	abnova, H00200316-A01	
		A3G	serum from a rabbit immunized with A3G peptide, CQDLSGRLRAILQNQEN	
